# Neck metastasis of the testicular teratoma in an adult: a case report

**DOI:** 10.1016/S1808-8694(15)30532-2

**Published:** 2015-10-18

**Authors:** Aziz Mustafa, Ilona Schwentner, Joachim Schmutzhard, Hannes Strasser, Georg M. Sprinzl

**Affiliations:** 1 M.D. (ENT specialist); 2 M.D. (ENT resident); 3 M.D. (ENT resident); 4 M.D. (Urologist); 5 M.D., Ph.D. (ENT specialist) ENT Clinic, University Clinical Centre of Kosovo, Prishtina, Kosovo 2) Department of Otorhinolaryngology, University Hospital, Innsbruck, Austria 3) Department of Urology, University of Innsbruck, Innsbruck, Austria

**Keywords:** neck dissection, neck metastasis, testicular teratoma

## INTRODUCTION

Testicular teratomas may present in both prepubertal and adult males. The prognosis differs greatly between these two groups. In children, teratomas most often occur before the age of four. They are seen in their pure form, and behave a benign behavior. In adults, teratomas are usually part of a mixed GCTs, and have the potential to metastasize. The presence of neck metastasis in patients with testicular germ cell neoplasms is a rare but well known phenomenon. The incidence of neck metastasis in testicular carcinoma has been reported to be present in up to 5% of the cases.^1^ Late relapses have been seen in many different locations, including the retroperitoneum, abdomen, pelvis, liver, mediastinum, lung, bone (femur, vertebra, and rib), lymph nodes outside the retroperitoneum and mediastinum, scrotum and inguinal regions, adrenal gland, chest wall, and buttocks.^2^.

## THE CASE

**Figure 1:**
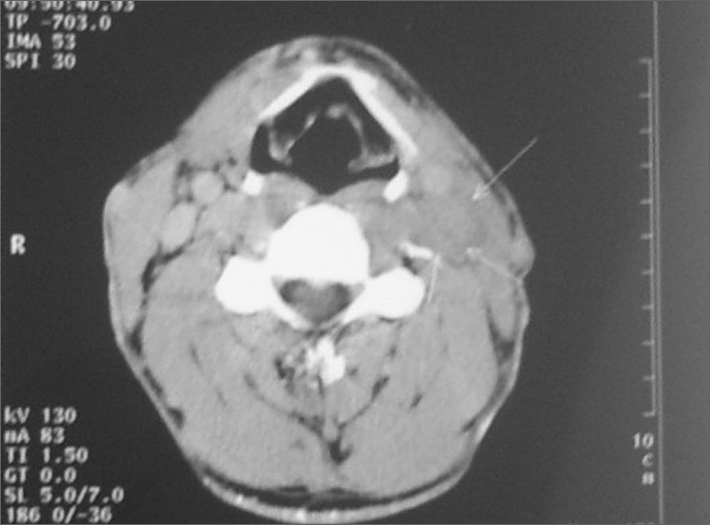
Enlarged conglomerate of cervical lymph nodes along the left jugular vein.

A 30 year old man was referred to our Department from Urological Clinic of the University Hospital, Medical University, Innsbruck, Austria. The patient had enlarged left supraclavicular lymph nodes, after surgical and chemotherapeutical treatment for retroperitoneal metastatic testicular teratoma. Six months before he was admitted to the Urology department, because of a painful swollen right testis. After CT-scan, immunohystochemistry (positive tumor markers: α-fetoprotein-AFP and human chorionic gonadothropin-hCG) and pathohistological diagnostic procedures, the diagnosis of a testicular teratoma was established. After right testis semicastratio, three cycles of chemotherapy with cisplatine, etoposide and bleomycin were performed. The patient suffered from a prolonged agranulocytosis during the last cycles of chemotherapy. Four months later, the control CT-scans detected suspicious metastatic lymph nodes of the retroperitoneal region. Retroperitoneal laparoscopic lymph node dissection was performed. The histopathological results showed an infiltration of bilateral para-aortal lymph nodes with testicular teratoma. The control CT scans after one month detected enlarged conglomerate of cervical lymph nodes along the left jugular vein (Figure 1.). Selective neck dissection of the regions II-VI was performed. All lymph nodes were removed and sent for pathological examination. The histopathological findings revealed metastatic testicular teratoma: germ cells in the seminiferous tubules, atypical, with occasional cells showing enlargement or multinucleation.

## DISCUSSION

Testicular germ cell tumours are rare neoplasm. They account 1% of all cancers in males. Their peak incidence is between the age of 25-35 years. There is a distinctive geographical and racial variation. The highest incidence is among white men in northern Europe. Both genetic and environmental factors are important in the development of testicular germ cell tumours. The age distribution suggests that an initiating event occurs prenatally and that the tumour develops until adolescence. The histopathology of testicular cancers is complex and the most important discrimination is between seminomas, which account for about 50% of the total, and teratomas or non-seminomatous germ cell tumours. Furthermore mixed tumours can be found in about 10% of the testicular neoplasms.^3^

The supraclavicular region of the neck is one of the possible places where testicular teratomas can metastasize2,3 Fine needle biopsy aspiration cytology (FNAC) presents a useful procedure in cases of metastatic cervical lymph nodes of GCTs, but the use of FNAC for the diagnosis of the primary testicular tumor is not advocated.^4^ In our case the FNAC was not used. The diagnosis of the neck metastasis was established in the base of imaging procedures.

The introduction of cisplatin-based chemotherapy for treatment of testicular GCTs led to dramatic improvement in the survival of patients with these neoplasms. Modern chemotherapy (PEB protocol: cisplatin, etoposide and bleomycin), together with surgical excision of primary tumor and metastases, results in survival in more than 90% of patients with non-seminomatous testicular GCTs (NSGCT). Because most relapses occur during the first two years after diagnosis, recurrence after two years of complete remission is considered rare.^2^

Weisberger and McBride showed that patients suffering from neck metastasis of NSGCTs have a surprisingly favourable prognosis if treated with PEB protocol chemotherapy and a specific technique of modified neck dissection. The levels of serologic tumor markers (hCG and AFP) have a high correlation with the presence or absence of residual malignant germ-cell tumor components. Surveillance for recurrence is facilitated by the availability of serologic tumor markers.^5^
